# My Experience with a Carbuncle (Anthrax.)

**Published:** 1872-01

**Authors:** Wm. A. Greene

**Affiliations:** Americus, Georgia, Vice-President of the Georgia Medical Association


### MY EXPERIENCE WITH A CARBUNCLE (ANTHRAX.)

BY WM. A. GREENE, M.D., AMERICUS, GEORGIA,
Vice-President of the Georgia Medical Association.

Many persons will no doubt think this a very ordinary and simple subject for a communication to a leading medical journal, but after carefully reading what I have to say, and upon maturer reflection, will arrive at a different conclusion, and, if they themselves have ever been the unfortunate victims of the disease, will agree with me that its treatment, *correct* treatment, is a thing "devoutly to be wished for." I am sure, from personal experience, (having myself just been the subject of carbuncle), that the treatment of this *very distressingly painful* affection is not properly appreciated or understood by most of the profession; neither do they properly comprehend its dangers. I think it would be "a good joke" for every Doctor to have a carbuncle early in his professional life, for the good of "suffering humanity;" for without this, I can truly say that no one ever properly appreciates the cries of agony from a carbuncular patient.

As intimated above, it has been my misfortune (but I hope the good fortune of some patient) to be afflicted during the past ten days with a severe carbuncle in the *very* middle of the calf of my right leg, which entirely disabled me. I have many complaints to file against this carbuncle. In the first place, it traveled out of its *usual* course in attacking *me*, for I am sure I am not a *subject* for its cruel infliction. I am not an old man—neither am I a feeble or diseased man—nor am I an intemperate man these latter days. And again, the *point* of attack was a singular one, for the integument on the back, from the nape of the neck to the pelvis, seems most subject to the disease; but, *fice-like*, it came up unawares and caught me by the leg.

But to the treatment of this disease I propose, briefly, to offer a few scattering thoughts.

The ordinary manner in which carbuncles were formerly treated, and still are by many, consists mainly in making large incisions through them, giving large quantities of food and stimulants, as well as considerable doses of quinine, bark and other stimulants—which treatment 1 think quite useless, and a source of the greatest discomfort to the patient. The plan usually recommended is to make two incisions crucially from border to border, and some say go even beyond the edges of the carbuncle into the adjacent healthy textures. It is said these incisions, if made when the carbuncle is only two or three days old, will prevent its spreading. But, as Mr. Paget very properly says, " even therein is a fallacy, for there is no sign by which, in looking at a commencing carbuncle, you can tell whether it will spread or not—whether it will have a diameter of an inch, or of three, six or ten inches." And further, you are not certain, in this early stage, whether you have a *carbuncle* or a *boil*, for the former is described as "a boil in an aggravated form." Every one has seen carbuncles spread when the first incisions have been made, and cut in the most approved fashion in depth and length and width. I say, then, that carbuncles will spread, after cutting, in as large a proportion of cases as without cutting. I acted on this principle in my own case, and am pleased with the result.

Again, it is said they are relieved of the *pain* by cutting. This is only partially true. A carbuncle of two or three days' standing, which is hard, tense and brawny, is very painful, and cutting, in some cases, *may* relieve a portion of the pain; but after this, when a carbuncle begins to soften, and pustules begin to form upon its surface and pus in its interior, it ceases to be very painful of its own accord, and without incisions; and if divided in this latter stage, what pain may exist is altogether unaffected by the cutting. My conclusion, then, is—if they can be cut in the first three or four days, while hard and brawny, it *may* relieve some measure of the suffering; at a later period, incisions have no influence at all.

Another point is, that by cutting carbuncles, you accelerate their healing, giving facility for the exit of sloughs. But herein is the greatest fallacy of all. Mr. Paget says: "When the cutting of carbuncles was more customary in this hospital than it is now—when I did not cut them, and some of my colleagues did—I used to be able to compare the progress of cases cut and of cases uncut, and time after time it was evident that the cases uncut healed more rapidly than those cut." My own case confirms this opinion of Mr. Paget's. I had no cutting done, and yet my carbuncle (a bad one) passed rapidly through its several stages in ten or twelve days. If I had followed the practice of incisions, I should have had to make a cut in one direction of about two inches, and in the other about one and a half inches; and after that I should have had not only the wounds—wide, open and gaping, and having themselves to heal—but a great part of the substance of the carbuncle fairly exposed, and also under the necessity of healing. But as it is, the whole of the space that now remains to heal is a series of openings in the middle of the carbuncle, gradually forming into one, through which the sloughs are being separated, nearly all being discharged, and which now merely remains to be healed like the cavities of ordinary abscesses. In this way, I narrowed greatly the extent of wounded surface to be healed. In my case, neither the whole carbuncle or its base sloughed. Carbuncles frequently suppurate about their centres, and slough only in their central parts, and the borders merely clear up by the softening and dispersion of the inflammatory products in them. Nay, in some cases, carbuncles completely abort. Every physician has seen this in his practice. Hence it seems to me that crucial incisions do not prevent extension—that it is only a limited number of cases in which the incisions diminish pain—

and that with regard to the time that is occupied in healing with or without incisions, the healing without incisions is very clearly and certainly a great deal quicker. I do not mean to say there are no conditions in which they are not useful. Sometimes a carbuncle sloughs in its central part, with one continuous slough of integument, holding in a quantity of pus. In such a case, you would cut through the slough, or through an adjacent part of the carbuncle, to let out the pus, as you would open an ordinary abscess. You ask, "What, then, is the treatment?" Mr. Paget reduces it to what commonly passes by the name of "doing nothing." And yet a good deal is done. His patients are carefully fed, washed, cleaned and bedded, and the carbuncle skillfully dressed and washed with proper things, and every care taken to shut out all untoward influences from them. In local treatment, he recommends, if the carbuncle is small, to cover it with emplastrum plumbi spread upon leather, with a hole in the middle, through which the pus can exude and the slough can come away. This, occasionally changed, he says, is all the covering a small carbuncle will need. For large ones, he thinks the best application is the common resin cerate. This should be spread large enough to cover the whole carbuncle, and over it laid a poultice of half linseed-meal and half bread. Now, if you want to exercise your skill, learn to make this poultice well, put it on well, and to keep it in its place. As the sloughs discharge, in the progress of the carbuncle, the cavities must be filled up with soft substances spread with this cerate. They should be carefully washed with carbolic acid or Cendey's fluid, or some deodorizing substance, and the cavities, if necessary, syringed out with it. Plenty of fresh air and good living complete the treatment.

In my own case, I departed somewhat from this line of treatment. The carbuncle first attracted my attention on Saturday, November 25th. Feeling a small, tender pimple on the calf of my leg—which, by next evening, was quite painful, and by Tuesday locomotion quite difficult—I had my friend, Dr. Coooper, to examine it, and the conclusion was that it was only an ordinary phlegmon. But Wednesday evening, November 29th, we were convinced that it was a carbuncle, and I went to bed and began treatment. I first applied equal parts of tincture of iodine, spirits of turpentine and glycerine over the inflamed borders, and covered the application with a poultice made of linseed-meal and bread. Thursday evening, the 30th, three points of suppuration were distinctly observed, and the base decidedly enlarged. Friday, December 1st, continued treatment, the carbuncle *looming*. Saturday, suppuration in full operation, and Sunday, line of demarcation very distinct, when carbolic acid gr. xii., pure glycerine ℥j., m., was freely appied with soft brush, and the poulticing continued. Monday morning, the 4th, I filled the spaces among the sloughs with permanganate potass gr. xv., water ℥ss., m., by means of a camel's-hair brush, and saturated a piece of lint with the same solution, and placed over the carbuncle, covering this with a dry piece of lint, and covered the whole with a bandage, and left it undisturbed for twenty hours, when the dressing was removed, and with it came a large slough, leaving a cavity large enough to hold *comfortably* a partridge egg. I filled this with lint saturated with the carbolic acid solution, and dressed the hole with benzoated zinc ointment spread on lint, which was not opened until Tuesday, December 5th, at noon. The slough was entirely discharged, leaving an ugly, deep excavation in my leg, which, I am thankful to state, is to-day (the 11th) nearly filled with healthy granulations, and I am on light duty. I continued the application of carbolic acid, as above, during the balance of the treatment, together with the benzoated zinc ointment as a dressing—to which I owe my rapid convalescence. This large carbuncle, I am sure, did well, and could not have been benefitted by the use of the knife in any particular.

In closing this hasty paper, written during the intervals of carbuncular pain and the application of poultices, etc., I must say that, in my opinion, there is no remedy in the *Materia Medica* equal to the permanganate of potass, in the very commencement of the suppurating stage of carbuncle, and continued until the slough is discharged. It is nearly painless, and in fact produces comfort after a few moments of applications, and there is no necessity of so frequently changing the dressing as in any other local treatment. I consider it almost a specific in this disease. After the slough is discharged, the carbolic acid, used in such strength as *not to be painful*, twice in twenty-four hours, is indicated; and there is nothing more efficacious. The benzoated zinc ointment, spread on a piece of soft lint and laid over the carbuncle thus dressed, completes the local treatment. This treatment has the further recommendation of being neat, clean and comfortable.

I hope this experience and treatment of carbuncle will not prove altogether uninteresting and uninstructive to your numerous readers; and if I succeed in saving a single poor human creature from the barbarous usage of "incising carbuncles," I shall feel not only amply repaid for my trouble, but that I have really done "suffering humanity" a great service.

